# Backbone and side chain ^1^H, ^13^C and ^15^N chemical shift assignment of the cargo-recognition domain MOTH of human TANGO1

**DOI:** 10.1007/s12104-026-10278-1

**Published:** 2026-09-11

**Authors:** Jelena Auch, Anna Carlotta Peddinghaus, Raphael Stoll

**Affiliations:** 1 Faculty of Chemistry and Biochemistry, Biomolecular Spectroscopy, RUBiospec|NMR, and PhenomeCentre@RUBUAR, Ruhr University of Bochum, Bochum, Germany; 2 Leibniz-Institut für Analytische Wissenschaften – ISAS – e.v, Dortmund, Germany

**Keywords:** NMR, Assignments, Protein transport, TANGO1 protein, MOTH domains

## Abstract

The cellular export of large-sized proteins, such as collagen or fibrillin, necessitates a specialized transport pathway tailored towards their size. For collagen and possibly also other bulky proteins, the Transport ANd Golgi Organization 1 (TANGO1) protein plays a substantial role in the transfer from the Endoplasmic Reticulum (ER) to the Golgi apparatus. TANGO1 and related proteins organize the ER exit sites and generate large export structures within the cytosol while anchored to the ER membrane. In contrast to other proteins involved, TANGO1 features a folded domain in the ER lumen, which seems to be central for detecting cargos. This cargo-recognition domain has been shown to bind the collagen-specific chaperone HSP47 (Ishikawa et al. 2016) as well as type IV collagen directly (Arnolds and Stoll 2023). However, the exact molecular mechanism of these interactions remains unknown. The resonance assignments presented here lay the foundation for the experiments studying the binding process on a molecular level and is designed to facilitate investigations into how TANGO1 detects and selects the cargos to be exported.

## Biological context

Collagen is the most abundant protein in animals and one of the main components of the extracellular matrix (ECM). Due to its scaffolding function, dysfunctions of collagen or malfunction of its biosynthesis and secretion leads to a wide range of diseases, for example brittle bones, dwarfism or fibrosis (Myllyharju and Kivirikko 2001; Tan 2024; Guo et al. 2025). One crucial step is the export from the Endoplasmic Reticulum (ER) to the Golgi apparatus and within this context the Transport ANd Golgi Organization 1 (TANGO1) protein has been identified as a key factor (Bard et al. 2006; Saito et al. 2009; Wilson et al. 2011). It consists of 1905 amino acids and is embedded in the membrane at the ER exit sites, with one part located in the cytosol and another in the ER lumen. TANGO1 features two coiled-coil domains and a proline-rich domain in the cytosol as well as a cargo-binding domain (CBD) in the ER lumen. Within the membrane, TANGO1 assembles into rings – together with related proteins (TANGO1S, cTAGE5) – through the interaction of their coiled-coil domains and organizes the formation of large, possibly tubular, carriers (Saito et al. 2009, 2011, 2014; Raote et al. 2017; Maeda et al. 2017). This specialized mechanism facilitates the export of collagen and potentially other scaffolding ECM components, such as fibrillin or fibronectin. To enable export, TANGO1 binds procollagen directly or through the complex with the Heat Shock Protein 47 (HSP47), a collagen-specific chaperone (Saito et al. 2009; Ishikawa et al. 2016). The cargo-binding domain of TANGO1 belongs to a family of proteins which all share the same functional fold, and for which we coined the name MOTH (MIA, Otoraplin, TALI/TANGO1 homology) domain. The core features an Src homology 3 (SH3) domain-like fold, which is elongated at both termini by additional β sheets. This appears to be a new functional fold as it has lost the ability to bind classical SH3 domain ligands. Recently it has been found (Arnolds and Stoll 2023) that TANGO1’s MOTH domain comprises more residues than previously assumed, which were not covered by the construct used so far (Ishikawa et al. 2016). To account for this and re-evaluate previous results, we here present the ^1^H, ^15^N and ^13^C assignment of the backbone and side chain chemical shift values of the full-length MOTH domain, using a construct comprising amino acid residues 21–151 of TANGO1. Our data (BMRB-ID 35043) is intended as an extension to the assignment of the shorter construct (BMRB-ID 34708). Furthermore, we determined the fast timescale dynamics on the ps to ns timescale by evaluating the ^15^N backbone relaxation rates per residue. This helps to identify those residues exhibiting chemical exchange, which could be relevant for the domain’s function.

## Methods and experiments

### Protein expression and purification

Since the cargo-binding domain of human TANGO1 was expressed and purified as published previously in detail (Arnolds and Stoll 2023), only the most important steps are outlined here. The sequence used corresponds to residues 21–151 of the full length human TANGO1, which is available in the Uniprot database (The UniProt Consortium 2025) under the Uniprot-ID Q5JRA6. Our construct contains additional N-terminal amino acids (Gly-His-Met) as remnants of the TEV cleavage site with which we removed the expression tag. These artificial residues are not considered in the assignment statistics and data analysis.

For expression, a modified pET19b vector with an N-terminal His_6_-tag and a TEV-cleavage site was transformed into BL21(DE3)RIL cells. The expression was performed in M9 minimal medium containing ^13^C glucose (4 g/L) and ^15^NH_4_Cl (3 g/L) as the sole carbon and nitrogen sources. The expression was performed at 30 °C overnight. The cells were harvested and lysed by microfluidization (buffer: 50 mM Tris/HCl, 1 mM EDTA, pH 8). The inclusion bodies obtained were washed (50 mM Tris/HCl, 1 mM EDTA and 1.5 vol% Triton-X-100) and subsequently solubilized (6 M guanidinium chloride, 12.5 mM NaHCO_3_, 87.5 mM Na_2_CO_3_, 0.2 M DTT, pH 10) overnight at room temperature. The pH was adjusted to 3 and after filtering and centrifugation, the pellets were resuspended (3 M guanidinium chloride, 4.7 mM sodium citrate dihydrate, 45.7 mM citric acid, pH 3). The solution was added dropwise into the refolding buffer (1 M L-arginine, 50 mM Tris/HCl, 1 mM EDTA, 0.5 mM oxidized glutathione, 2.5 mM reduced glutathione) at 8 °C. For subsequent concentrating and rebuffering (PBS, pH 7) the ÄktaFlux system (3 kDa MWCO cartridge, GE Healthcare) was used. The His_6_-tag was cleaved at 30 °C for 3 h and the digested proteins were separated by Protino-Kit Ni-TED 2000 columns (Machery-Nagel). Subsequently, the protein was further purified by size-exclusion chromatography using a HiLoad 26/600 Superdex 75 pg column (Merck).

### NMR samples and spectroscopy

Protein samples were measured in PBS (pH 7.4) containing 2% protease inhibitor cocktail (Roche, cOmplete™ Ultra Mini Tablets) and DSS as reference. If an amide proton is required for the experiment, the sample contained 10% D_2_O, otherwise the sample was lyophilized and dissolved in 100% D_2_O. The protein concentration was adjusted to 1 mM for the experiments used for resonance assignment and to 0.75 mM for the relaxation rate experiments.

For the assignment of the backbone and side chain chemical shift values of hsTANGO1 (21–151), a set of triple-resonance spectra and NOESY spectra were recorded, the latter with an NOE mixing time of 120 ms. A detailed list of experiments and settings can be found in Table 1. The experiments for the determination of the *R*_1_ and *R*_2_ relaxation rates were measured as pseudo-3D spectra with an interscan delay of 5 s. For the *R*_1_ determination relaxation delays of 5 ms, 60 ms, 100 ms, 200 ms, 400 ms, 600 ms, 800 m, 1200 ms, 1800 ms, 2400 ms were used and for the *R*_2_ determination the following number of CPMG cycles were set: 0, 1, 2, 4, 6, 8, 10, 12, 14. For error estimation, two *T*_1_ delays and three numbers of CPMG cycles for *T*_2_ were measured in duplicates.

All spectra were recorded at 310 K on a Bruker AVANCE III HD 700 spectrometer. The experiments for determining relaxation rates were additionally recorded on a Bruker AVANCE NEO 600 spectrometer. TopSpin 4.2.0 (Bruker BioSpin GmbH 2023) and CcpNmr Analysis 3.1.1 (Skinner et al. 2016) were used for processing and spectra analysis, respectively. The latter was also used for calculating the assignment statistics. The *R*_2_ and *R*_1_ relaxation rates were analyzed using the software RELAX (d’Auvergne and Gooley 2008a, b; Bieri et al. 2011) on the NMRbox suite (Maciejewski et al. 2017).

**Table 1:** NMR experiments and parameters used for chemical shift assignment and relaxation rate determination

Spectrum	Pulse program (Bruker BioSpin GmbH)	NS	NUS %	Number of data points / TD			Spectral width / ppm		
				F1	F2	F3	F1	F2	F3
^15^N-HSQC	hsqcfpf3gpphwg	32	-	128 N	2048 H	-	25.00	15.94	-
^13^C-HSQC	hsqcctetgpsp	16	-	256 C	1024 H	-	80.00	12.99	-
HNHA	hnhagp3d	32	25%	40 N	128 H	2048 H	25.00	14.00	15.94
HNCA	hncagpwg3d	32	25%	128 C	48 N	2048 H	37.60	25.00	15.94
HNCO	hncogpwg3d	32	25%	128 C	40 N	2048 H	12.00	25.00	15.94
HNcaCO	hncacogpwg3d	32	25%	128 C	40 N	2048 H	12.00	25.00	15.94
HNCACB	hncacbgpwg3d	64	25%	128 C	40 N	2048 H	80.00	25.00	15.94
CBCAcoNH	cbcaconhgpwg3d	32	25%	128 C	40 N	2048 H	80.00	25.00	15.94
HCCH-TOCSY	hcchdigp3d	16	25%	128 H	64 C	2048 H	14.00	80.00	14.00
HCCH-COSY	hcchcogp3d	16	25%	128 H	64 C	2048 H	14.00	80.00	14.00
HCCcoNH	hccconhgpwg3d3	32	25%	128 C	40 N	2048 H	80.00	25.00	15.94
HcccoNH	hccconhgpwg3d2	64	25%	128 H	40 N	2048 H	15.94	25.00	15.94
hbCBcgcdHD	hbcbcgcdhdgp	512	-	64 C	2048 H	-	40.00	15.94	-
hbCBcgcdceHE	hbcbcgcdcehegp	512	-	64 C	2048 H	-	40.00	15.94	-
^15^N-HSQC-NOESY	noesyhsqcf3gpwg3d	32	-	128 H	40 N	2048 H	15.94	25.00	15.94
^13^C-HSQC-NOESY	noesyhsqcetgp3d	32	-	128 H	64 C	2048 H	14.00	80.00	14.00
^15^N *T*_1_ relaxation	hsqct1etf3gpsi3d	32	-	-	128 C	2048 H	-	29.40	15.94
^15^N *T*_2_ relaxation	hsqct2etf3gpsi3d	32	-	-	128 C	2048 H	-	29.40	15.94

### Extent of assignment and data deposition

In this study we present the chemical shift values of the backbone and side chain ^1^H, ^13^C and ^15^N resonances of the cargo-binding domain MOTH of human TANGO1 (21–151). The assigned resonances can be accessed *via* the Biological Magnetic Resonance Data Bank (BMRB) under the accession number 35,043.

**Fig. 1:**
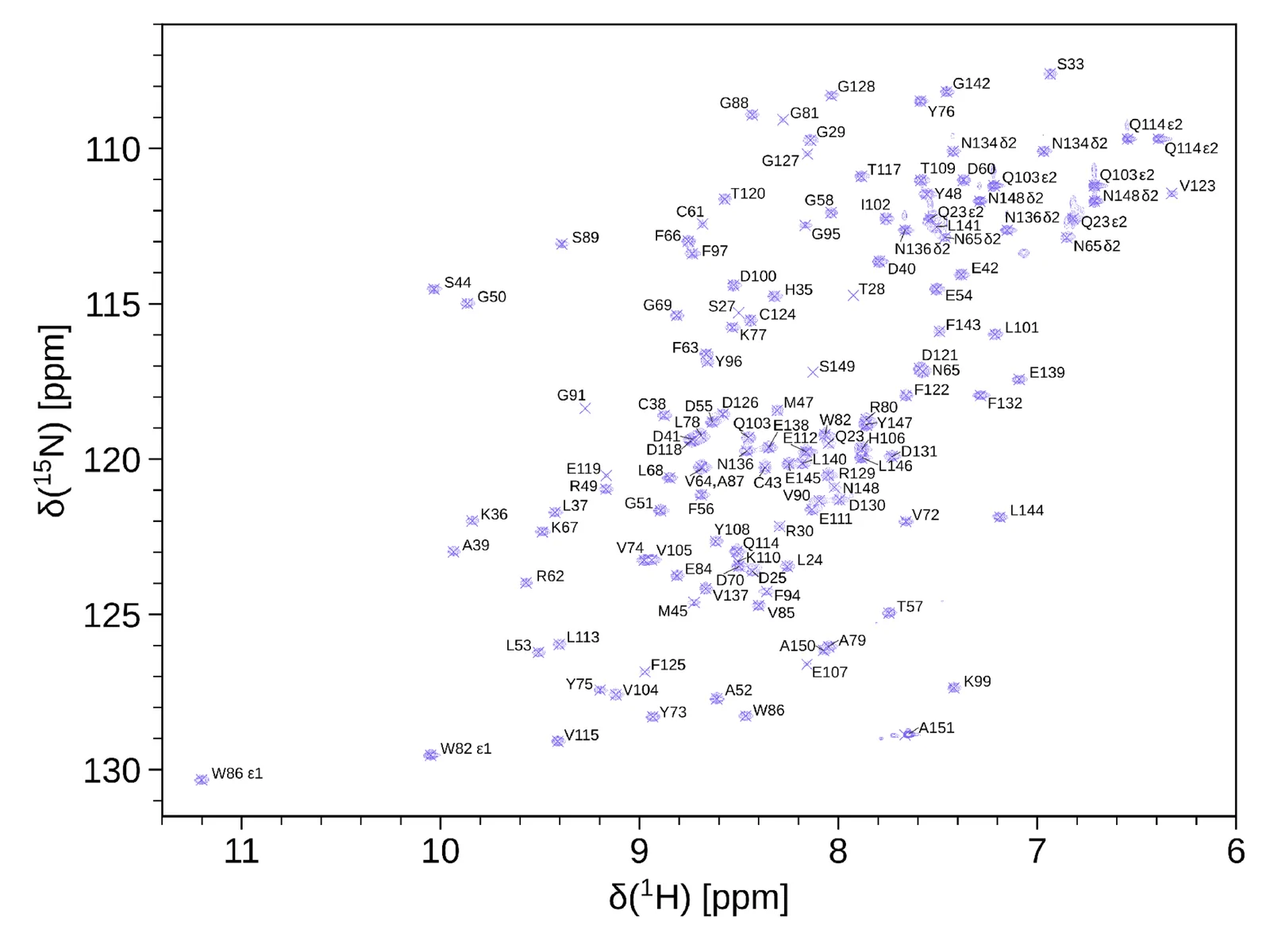
Assigned ^1^H–^15^N-HSQC spectrum of residues 21–151 of human TANGO1 in PBS buffer (pH 7.4). The spectrum was recorded at 700 MHz and at 310 K

Excluding prolines, 124 backbone amide resonances are expected in the ^1^H–^15^N-HSQC spectrum (Fig. 1). We observed 116 signals and assigned 114 residues, which represents 91.1% of the expected backbone resonances. Unassigned residues are G22, R31, F32, E34, L46, R92, T93, H133, N134, and Y135. The assignment percentage is 92.4% of CO, 97.7%/96.5% of C_α_/H_α_ and 97.5%/95.8% of C_β_/H_β_. The overall completeness of the side chain resonance assignment is 81.0%. The analysis of the C_β_ and C_γ_ chemical shift values of the prolines revealed that one out of seven prolines, Pro83, adopts a *cis*-conformation according to the reference values (Schubert et al. 2002).

**Fig. 2:**
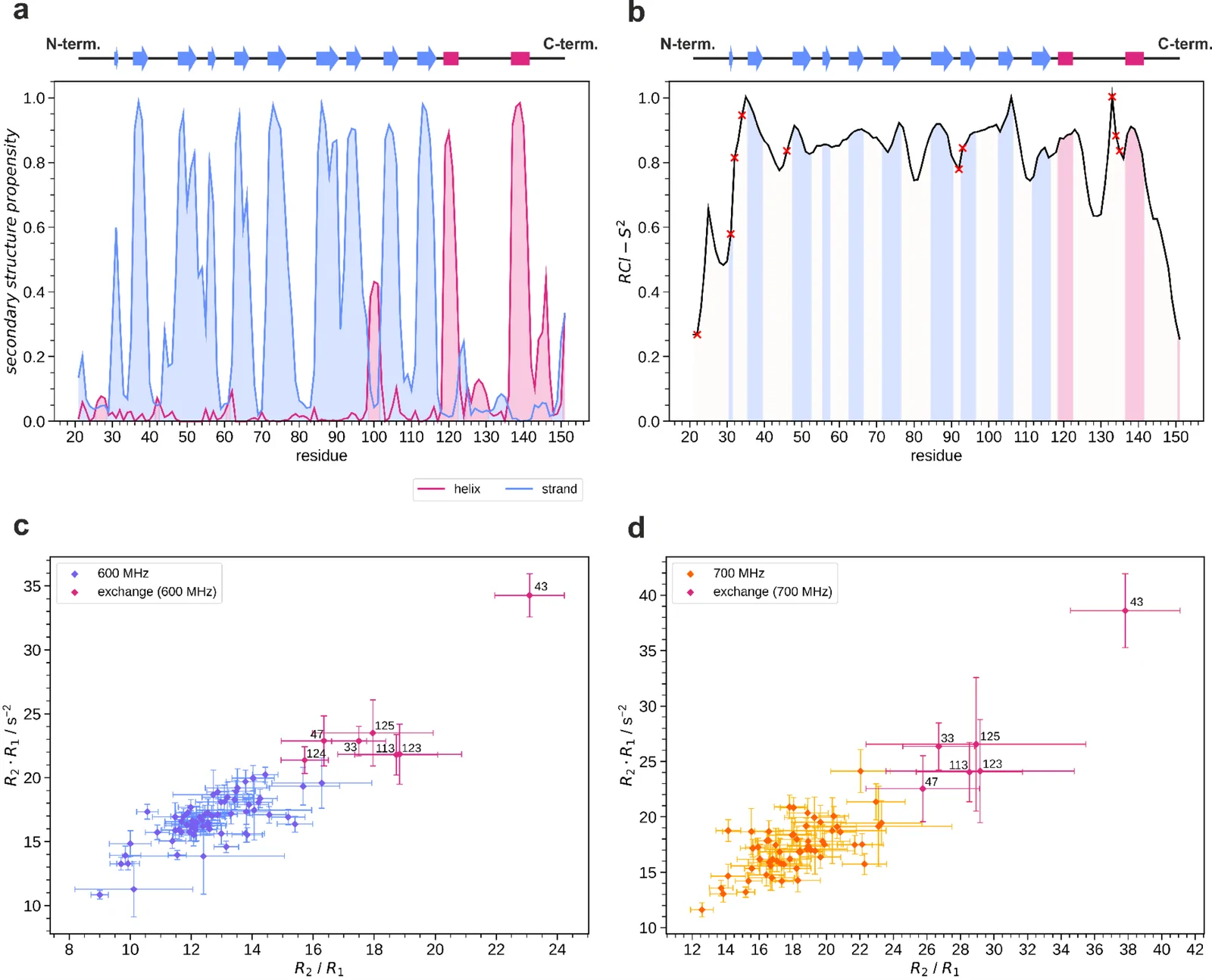
**a** Secondary structure propensity plot and cartoon representation, calculated using the TALOS-N webserver, with β sheets displayed in blue and helices in pink. **b** Random Coil Index derived order parameter (*RCI-S*^2^) per residue, calculated using the TALOS-N webserver. Residues without amide ^1^H–^15^N resonance assignment are marked by red crosses. The colors below the curve illustrate the most probable secondary structure determined for this residue based on the secondary structure propensity, with β sheets displayed in blue and helices in pink. **c**/**d** Product of longitudinal and transversal relaxation rates, *R*_1_ and *R*_2_, plotted against their ratio at 600 MHz (**c**) and 700 MHz (**d**). Residues for which both values are greater than the mean plus the standard deviation are labelled by their residue number and highlighted in pink

The obtained chemical shift values were used for predicting the secondary structure propensity per residue with TALOS-N (Shen and Bax 2013) (Fig. 2a). The predicted secondary structure motifs are in good agreement with the structure in solution of residues 21–131 of TANGO1 (PDB-ID 7R3M). This indicates that the structure of the domain’s core is not likely to be fundamentally different for the construct comprising residues 21–151. The C-terminal elongation features α-helices for residues 119–123 and 137–141 according to the TALOS-N prediction. This confirms that the construct using residues 21–121 does not cover the entire folded domain. Furthermore, we calculated the Random Coil Index order parameter *RCI*-*S*^2^ per residue with TALOS-N (Shen and Bax 2013) (Fig. 2b). The order parameter values agree with the predicted secondary structures, showing increased *RCI*-*S*^2^ values for secondary structures and reduced *RCI*-*S*^2^ values for loops. Residues with an order parameter below 0.6 are considered to be highly flexible, as in the case for the N- and C-terminus. A slightly increased flexibility compared to the secondary structures is also observed in the region between the helices, which may be the reason as to why the end of the domain was initially thought to be located around residue 121.

To identify those residues that may undergo chemical exchange, the longitudinal and transversal relaxation rates *R*_1_ and *R*_2_ of the amide ^15^N were determined at 600 MHz and 700 MHz. Increased values of both, the ratio (*R*_2_/*R*_1_) and product (*R*_2_ · *R*_1_) of the relaxation rates can be used as an indicator of chemical exchange (Fig. 2c and d) (Kneller et al. 2002). This analysis identified residues Ser33, Cys43, Met47, Leu113, Val123, and Phe125 as candidates, as they fulfill the condition at both frequencies. It is not yet known as to whether this exchange process is correlated with any function. The resonance assignments presented here serve as the basis for further NMR (exchange) experiments to elucidate the mechanism by which TANGO1’s cargo-recognition MOTH domain is able to select which cargos are to be exported.

## Data Availability

All assigned chemical shift values of residues 21-151 of human TANGO1 have been deposited in the Biological Magnetic Resonance Data Bank (BMRB) under the accession number 35043.
